# Internet Attachment–Based Compassion Therapy for Adults With Chronic Medical Conditions: Randomized Controlled Trial

**DOI:** 10.2196/86679

**Published:** 2026-08-11

**Authors:** Rocío Herrero, Marian Martínez-Sanchis, Ángel Zamora, María Dolores Vara, Daniel Campos, Javier García-Campayo, Rosa Baños

**Affiliations:** 1 Department of Basic, Clinical, and Psychobiology Psychology, Universitat Jaume I, Castellón, Castellón, Spain; 2 Instituto de Salud Carlos III, Spanish Biomedical Research Centre in Physiopathology of Obesity and Nutrition, Av. Monforte de Lemos, 3-5, Madrid, 28029, Spain, 34 616773277; 3 Department of Psychology, Faculty of Health Sciences, Universidad Europea de Valencia, Valencia, Spain; 4 Aiglé Valencia, Valencia, Spain; 5 Polibienestar Research Institute, Universitat de València, Valencia, Spain; 6 Department of Personality, Evaluation, and Psychological Treatments, Universitat de València, Valencia, Valencia, Spain; 7 Departamento de Psicología y Sociología, Facultad de Educación, Universidad de Zaragoza, Zaragoza, Spain; 8 Instituto de Investigación Sanitaria Aragón, Zaragoza, Aragon, Spain; 9 Red de Investigación en Cronicidad, Atención Primaria y Prevención y Promoción de la Salud, Madrid, Spain; 10 Department of Medicine, Psychiatry and Dermatology, Universidad de Zaragoza, Zaragoza, Spain

**Keywords:** internet-based intervention, chronic disease, quality of life, self-compassion, randomized controlled trial

## Abstract

**Background:**

Chronic medical illnesses coexist with mental health challenges, negatively impacting quality of life and well-being. Compassion-based interventions have shown promise for individuals with chronic conditions, yet accessibility barriers limit their implementation. Internet-delivered formats may address these limitations while maintaining effectiveness. To our knowledge, no fully self-guided, internet-delivered attachment-based compassion intervention has been tested in a transdiagnostic chronic illness population.

**Objective:**

This study aimed to evaluate the efficacy of internet attachment-based compassion therapy (iABCT) in improving quality of life and well-being (primary outcomes), and secondary psychological variables in adults with chronic medical conditions, compared to a waiting list (WL) control, and to assess acceptability and implementation barriers.

**Methods:**

A 2-arm, parallel-group, exploratory randomized controlled trial was conducted in Spain, with open-access recruitment through online and offline channels, and assessments completed via web-based self-report questionnaires. Adults (≥18 years) with a chronic medical condition and ability to read Spanish were eligible; individuals with terminal illness, current psychotherapy, or severe mental disorders were excluded. Participants were randomly allocated (1:1) to iABCT or a WL control (no intervention for 3 months) using a stratified, centralized computer-generated sequence. iABCT is a fully self-guided, 8-module online program delivered over 8 weeks, grounded in attachment theory and compassion-focused principles. Blinding of participants was not possible due to the nature of the intervention. Primary outcomes were quality of life (EQ-5D) and well-being (Pemberton Happiness Index [PHI]), assessed at baseline, 3 months, and 6 months. Data were analyzed using linear mixed-effects models under an intention-to-treat approach.

**Results:**

A total of 146 participants were randomized (iABCT n=72; WL n=74), all included in intention-to-treat analyses, with data available for 27 iABCT and 26 WL participants at 3-month follow-up. Between-group comparisons favored iABCT for overall quality of life (Cohen *d*=0.62, 95% CI 0.07-1.17) and preoccupied attachment, while well-being and most secondary outcomes showed within-group improvements without significant between-group differences. Quality of life improvements were maintained at 6 months within the iABCT group. No serious adverse events were reported in either group. Participants reported high satisfaction and usability. Qualitative interviews identified lack of therapist contact and insufficient monitoring as main engagement barriers.

**Conclusions:**

This trial provides preliminary evidence that iABCT may improve quality of life in adults with chronic medical conditions. It represents the first fully self-guided, internet-delivered attachment-based compassion intervention tested across diverse chronic conditions, uniquely integrating attachment security as a theoretical change mechanism alongside self-compassion—an approach that may address access barriers related to geography, mobility, or socioeconomic constraints. However, findings remain exploratory given high attrition, modest sample size, and no active control. Future adequately powered trials incorporating minimal human support, active control conditions, and cost-effectiveness analyses are needed to optimize real-world implementation.

## Introduction

### Background and Rationale

Chronic illnesses are typically characterized by their duration, lasting at least 1 year, and by the ongoing need for medical care or limitations they impose on daily functioning [1]. Recent epidemiological data from a large-scale study identified obesity (19%), hypertension (13%), diabetes mellitus (9%), esophagitis-gastritis (5.5%), and thyroid disease (5.3%) as the 5 most prevalent chronic conditions, with significant variations in age and sex distribution across different conditions [2]. Given their increasing incidence and considerable impact, these health issues impose substantial burdens on patients, their families, and broader societal structures. In fact, chronic diseases are responsible for approximately 90% of annual health care spending in the United States, amounting to US $4.1 trillion [1].

### Chronic Illness and Mental Health Comorbidities

The widespread coexistence of mental health issues with chronic physical illnesses poses one of the greatest challenges to global health care systems [3]. These complex comorbid relationships have been comprehensively documented in the scientific literature [4], with substantial evidence demonstrating that patients with chronic physiological pathologies (including persistent pain syndromes, diabetes mellitus, and inflammatory gastrointestinal disorders) frequently manifest concurrent psychopathologies, particularly depressive and anxiety disorders [5-7]. These mental health comorbidities are commonly linked to worse health outcomes, such as reduced life satisfaction and lower overall quality of life [4,8,9].

The presence of comorbidities suggests a bidirectional relationship, where the outcomes of the illness are shaped not only by its severity and progression but also by accompanying psychological dynamics. To illustrate, psychological adjustment, coping behaviors, and how patients manage their illness play a pivotal role in influencing physical health outcomes, including rates of morbidity, mortality, and complications [10-13].

### Psychological Interventions and Compassion-Based Therapies

Additionally, detrimental psychological processes (eg, illness-related shame, self-criticism, or rumination) constitute a barrier to implementing self-care behaviors involved in adaptive illness management (eg, treatment plan adherence, exercise, or adherence to dietary guidelines), which, in turn, could further increase distress, worsen prognosis, and diminish the quality of life [14,15]. Consequently, both self-care behaviors included in illness management as well as detrimental psychological processes have become a target of different psychological interventions aimed at promoting the quality of life of the population with chronic medical illness [16].

This is the case of compassion-based interventions (CBIs), which refer to psychological interventions aimed at enhancing compassionate and self-compassionate responses that involve the recognition of suffering and the inclination to relieve it with an act of kindness rather than criticizing, blaming, or pitying [17]. Fostering compassion and self-compassion may support illness management by encouraging acceptance of suffering as a fundamental human experience and promoting a gentler stance toward personal challenges [18].

Several individual studies have shown that CBIs can significantly enhance quality of life for individuals living with chronic health conditions [19-21]. This evidence has been corroborated by recent systematic reviews and meta-analyses, which concluded that CBIs produce improvements across multiple outcomes including depression, anxiety, self-compassion, and health-related quality of life [22-24]. In sum, being able to approach difficulties with a compassionate attitude helps individuals to feel empowered with new management strategies and promotes a sense of calm and agency to provide comfort to themselves, which facilitates the implementation of self-care behaviors and adaptive illness management [25,26].

### Attachment-Based Compassion Therapy

Among the various CBIs available, attachment-based compassion therapy (ABCT) represents a particularly promising approach that has shown effectiveness among both healthy individuals and those managing chronic medical illnesses [27]. This therapy is based on attachment theory [28], which provides a framework for understanding the links between close relationships and psychopathology and includes specific practices to identify and develop a secure attachment style to promote compassion for oneself and others [27]. Specifically, the ABCT has shown its efficacy and applicability for the treatment of fibromyalgia, showing improvements in psychological outcomes such as functional status [29] and biological outcomes [30].

Despite ABCT’s demonstrated efficacy, there are specific delivery barriers that could interfere with the effectiveness of CBIs in people with chronic illnesses, such as limitations of access, mobility, or transportation. In order to tackle these limitations and to respond to the growing need of health care systems for scalability and sustainability, evidence-based interventions can benefit from adapting their delivery format through information and communications technologies (ICTs) as a complementary or alternative delivery mode [31,32]. In fact, previous research supports the notion that the psychological outcomes of people living with chronic illnesses can be improved with a self-delivered online intervention [33,34], and recent evidence shows the potential value of delivering CBIs online in the context of chronic illnesses [35,36]. Moreover, other results show that adapting CBIs to an online format may improve adherence and facilitate the involvement of chronic patients in better management of their illness [32]. Regarding the ABCT approach, an online version of internet attachment-based compassion therapy (iABCT) has been developed to be totally self-applied over the internet for Spanish speakers. The iABCT is currently assessed in a feasibility study for the general population [37]. The iABCT is designed as a stand-alone, fully self-administered psychological intervention, though the original ABCT protocol has demonstrated utility both as an independent treatment and as a complement to usual care in clinical settings [38].

In conclusion, although CBIs delivered through the internet show promise as a cost-effective solution and initial research has explored the feasibility and acceptability of the iABCT in a Spanish general population [37], more research is needed on their efficacy specifically in the context of chronic medical conditions. While online interventions offer numerous advantages, recent studies have identified engagement challenges that may affect their efficacy. For instance, Finlay-Jones et al [35] found that despite significant improvements in well-being and distress outcomes, program engagement and retention rates were suboptimal in their web-based self-compassion intervention for young people with chronic conditions. These findings highlight the importance of examining potential barriers to engagement and treatment acceptability in online CBIs.

Given the preliminary nature of digital CBIs for chronic illness populations, a waiting list (WL) control was chosen as the comparator to establish the initial efficacy of iABCT before conducting comparisons with active treatment alternatives, following recommended sequential evaluation frameworks for behavioral interventions [39].

### Objectives

Therefore, this study aims to explore the efficacy of the iABCT to improve quality of life and well-being (primary outcomes) and other psychological variables (secondary outcomes) in a population with chronic medical illness, compared to WL, as well as examine treatment acceptability and implementation barriers and facilitators. We hypothesized as follows:

- H1: Participants receiving iABCT will show significantly greater improvements in quality of life and well-being at the 3-month follow-up compared to WL control, and these improvements will be maintained within the iABCT group at the 6-month follow-up.
- H2: Participants receiving iABCT will show improvements in secondary psychological outcomes at the 3-month follow-up compared to WL control, with improvements maintained at the 6-month follow-up.
- H3: The iABCT intervention will demonstrate high acceptability in terms of satisfaction, usability, and participant feedback.

## Methods

### Patient and Public Involvement

No formal patient or public involvement was undertaken in the design, conduct, or reporting of this trial.

### Trial Design

This study used a 2-arm, parallel-group randomized controlled trial (RCT) design to evaluate the efficacy of iABCT in individuals with chronic medical conditions. Participants were randomly assigned to either the intervention group (iABCT) or a WL control group. Assessments were conducted at baseline, 3-month follow-up, and 6-month follow-up. The WL group received access to the intervention after completing the 3-month assessment. As a result, between-group comparisons were feasible only at the 3-month follow-up, while 6-month assessments captured within-group maintenance for the iABCT group and postintervention effects for the WL group following delayed access.

The study adhered to the CONSORT (Consolidated Standards of Reporting Trials) 2025 statement [40]. The completed CONSORT checklist is provided in Checklist 1. The study also followed the CONSORT eHEALTH V1.6 guidelines [41], and the SPIRIT (Standard Protocol Items: Recommendations for Interventional Trials) statement [42].

### Changes to Trial Protocol

Two deviations from the preregistered protocol [38] occurred during trial implementation. First, 5 disease-specific illness interference measures (Diabetes Distress Scale, Roland-Morris Questionnaire, Revised Fibromyalgia Impact Questionnaire, Migraine Disability Assessment Questionnaire, and Inflammatory Bowel Disease Questionnaire) and the Illness Perception Questionnaire-Revised were included as secondary outcomes but are not reported as inferential analyses in this paper. Although data for these measures were collected and are available, subgroup sizes within each medical condition were small and highly unbalanced across groups (refer to Table S1 in Multimedia Appendix 1), precluding reliable statistical inference. Descriptive statistics are nonetheless reported in Table S1 in Multimedia Appendix 1 for transparency. Second, sensitivity analyses using multiple imputation methods, prespecified in the registered protocol, were not conducted. Given the substantial attrition rate observed across assessment points (ie, >30% missing data), additional imputation procedures were considered unnecessary. Instead, analyses were performed using mixed-effects models estimated with Restricted Maximum Likelihood (REML) procedures, which allow the inclusion of all available observations and provide unbiased parameter estimates under the missing-at-random (MAR) assumption, consistent with the result of Little MCAR test (*χ*²_153_=150.97, *P*=.53). Mixed-effects models are considered a robust and recommended approach for handling incomplete longitudinal data, particularly in repeated-measures designs with moderate to high attrition, as they avoid case-wise deletion and do not require prior imputation of missing values [43].

No major bug fixes, system downtimes, or content changes to the iABCT platform occurred during the trial period.

### Eligibility Criteria

Inclusion criteria were: (1) adults aged 18 to 70 years, (2) self-reported diagnosis of a chronic medical condition, defined as a diagnosed medical illness lasting at least 1 year and requiring ongoing medical care or imposing functional limitations (participants indicated their specific condition and its duration during screening); (3) ability to read and understand Spanish; and (4) access to a computer with internet connection and an email account. Exclusion criteria were (1) presence of a terminal disease, (2) presence of severe psychiatric disorder comorbidities (schizophrenia, substance dependence, bipolar disorder, or psychotic illness) or severe neurologic or medical condition; and (3) receiving psychological treatment or mindfulness training at the time of recruitment.

As the iABCT intervention was fully self-administered with no human care providers involved in its delivery, eligibility criteria for individuals delivering the intervention were not applicable.

### Settings and Locations

This was a fully web-based trial conducted entirely online with no face-to-face contact. Participants were recruited nationally through online and offline channels, including the study website, flyers posted on social media platforms (eg, Facebook [Meta Platforms, Inc], Instagram [Meta Platforms, Inc], and LinkedIn [LinkedIn Corp]), doctors’ referrals, and associations for people with chronic conditions. All distributed materials included a dedicated email address through which participants could contact the research team.

All study procedures, including eligibility screening, informed consent, intervention delivery, and outcome assessments, were completed remotely via the Psychology and Technology web platform [44], accessed using a computer with an internet connection (as specified in the inclusion criteria). PDF files of intervention materials were available for download to allow offline review. Outcomes were self-assessed by participants through online questionnaires administered at baseline, 3-month follow-up, and 6-month follow-up. The trial was conducted without institutional affiliations being prominently displayed during recruitment.

### Intervention and Comparator

#### Experimental Group: iABCT

The iABCT is a fully digital, self-guided adaptation of the original ABCT, developed for online delivery [37]. ABCT is grounded in attachment theory and incorporates compassion-focused meditative practices designed to foster awareness of—and, when appropriate, address—maladaptive attachment patterns, particularly those formed in early caregiver relationships [27]. In addition, it integrates structured exercises that cultivate both compassion and self-compassion, with the aim of enhancing psychological well-being and improving interpersonal functioning.

The iABCT intervention consists of 8 sequential modules delivered over an 8-week period (refer to Table 1 for detailed module content). Each module follows a consistent structure that includes (1) clearly defined learning objectives; (2) theoretical content aligned with the module’s central theme; (3) formal and informal experiential practices; (4) brief assessments to evaluate comprehension; (5) preparatory tasks to be completed before proceeding to the next module (ie, homework); and (6) a concise summary of key concepts. The content is delivered through a variety of multimedia formats, including written texts, images, illustrations, videos, interactive components, audio-guided meditations, and daily practical exercises, all designed to facilitate engagement and the integration of learning into daily life.

**Table 1.** Structure and contents of internet attachment-based compassion therapy.

Module	Theoretical component	Formal practice	Informal practice
0: Introduction to attachment-based compassion therapy	What is compassion? Contexts of application Attachment-based compassion therapy: structure and rationale Meditation and compassion: formal and informal practice Tips about meditation practice: when, where, how much, and how to meditate The importance of progressiveness in compassion and homework	—^a^	3-minute compassionate practice
1: Preparing ourselves for compassion. Kind attention	The workings of our brain The reality of suffering: primary and secondary suffering What is and is not compassion?	Compassionate breathing and compassionate body scan Compassion in coping with difficulties	Self-compassion diary Savoring and giving thanks
2: Discovering our compassionate world	Going deeper into compassion and mindfulness Compassion and related terms Fear of compassion	Connecting with basic affection Developing a safe place The compassionate gesture Identifying the figure of secure attachment	The object that joins us to the world (optional) Diary of compassion practice What are we good at?
3: Developing our compassionate world	How compassion works The figure of secure attachment Efficacy of compassion Self-criticism	Developing the figure of secure attachment Developing the compassionate voice	Writing a letter to the figure of secure attachment (optional)
4: Understanding our relationship with compassion	The biological bases of compassion Attachment styles Guilt Importance of these styles in everyday life	Becoming aware of our attachment style Ability to receive affection: friend, indifferent person, and enemy Guilty repair practice	Letter to your parents Observing our attachment styles in daily life
5: Working on ourselves	The importance of affection toward ourselves and others Embarrassment	Showing affection to friends and indifferent people Showing affection to ourselves Reconciliation with our parents Repairing embarrassment	The greatest display of affection (in general and from our parents) 3 positive aspects and 3 negative aspects of our parents
6: Understanding the importance of forgiveness	The concept of forgiveness Phases of forgiveness Utility of forgiveness Basic resistances to generate forgiveness Resources to generate forgiveness	Forgiving yourself Asking others for forgiveness (optional) Forgiving others and showing compassion to enemies	Interdependence Compassion in daily life
7: Consolidating the practice of compassion	Working in 3 periods (past, present, and future) Envy Usefulness of being our attachment figure Difficult relationships How to keep up the practice of compassion for a lifetime	Working with envy Becoming our own attachment figure Handling difficult relationships	Our values and their relationship with compassion What would our lives be like if we started over?

a Not applicable.

Additionally, minor adaptations were introduced to tailor the intervention to individuals with chronic conditions. Specifically, examples referring to living with a chronic illness were incorporated throughout the modules, and an additional exercise was included in the final module aimed at fostering illness acceptance.

Participants engage in compassion-focused meditations targeting various relational figures, including the self, close others, neutral individuals, and those perceived as difficult. The program also invites participants to explore their own attachment styles and to reflect on how these patterns influence their current interpersonal relationships.

Across modules, participants are gradually introduced to key concepts related to compassion, including its definition, underlying mechanisms, and applications within the context of illness. Emphasis is placed on developing a compassionate stance toward one’s suffering, with the goal of reshaping the individual’s approach to illness and promoting improvements in quality of life. In parallel, participants receive psychoeducational content that supports the recognition of personal attachment dynamics and encourages the development of healthier relationships with oneself and others.

Each module is intended to be completed in approximately 60 minutes, and the full intervention is structured to span an 8-week period. To enhance adherence and minimize attrition, participants who had not logged into the platform for 1 week received automated email reminders prompting them to continue their progress. The content and functionality of the iABCT platform were frozen for the duration of the trial; no updates or modifications were made to the intervention during the study period.

#### Control Group: WL

Participants assigned to the WL control group did not receive any intervention during the initial 3-month period following enrollment and completion of the baseline assessment. After completing the second assessment at the 3-month follow-up, individuals in this group were granted full access to the iABCT program.

Participants in both groups were not restricted from continuing to receive usual medical care for their chronic conditions throughout the trial. No data on concomitant medical or psychological treatments initiated during the trial period were systematically collected. Participants receiving current or past psychotherapy within the previous 12 months were excluded at screening (see the “Eligibility Criteria”).

### Outcomes

#### Primary Outcome Measures

##### Quality of Life

Assessed using the EQ-5D [45], which measures health-related quality of life across 5 functionality dimensions. The Spanish version demonstrates good reliability and validity [46]. In this study, Cronbach α was 0.70.

##### Well-Being

Measured with the Pemberton Happiness Index (PHI) [47], which assesses general, hedonic, eudaimonic, social, and experienced well-being. The Spanish validation shows strong psychometric properties. Cronbach α in this study was 0.90.

### Secondary Outcome Measures

#### Compassion and Self-Compassion

Evaluated using the Sussex-Oxford Compassion for the Self Scale (SOCS-S) [48], a 20-item measure with 5 subscales. A Spanish translation was used, with the total score yielding a Cronbach α of 0.92.

#### Self-Care Behaviors

Assessed with the Mindful Self-Care Scale - Brief version (B-MSCS) [49], a 24-item scale measuring frequency of self-care behaviors across 6 domains. The Spanish translation demonstrated Cronbach α ranging from 0.71 to 0.93 across domains.

#### Self-Criticism

Measured using the Self-Critical Rumination Scale (SCRS) [50], a 10-item questionnaire assessing self-criticism. The Spanish validation [51] demonstrated good psychometric properties. Cronbach α was 0.93.

#### Psychological Symptoms

Assessed with the Depression, Anxiety, and Stress Scale (DASS-21) [52]. The Spanish validation [53] has good psychometric properties. Cronbach α was 0.95.

#### Attachment Styles

Evaluated using the Relationships Questionnaire (RQ) [54], which assesses secure, preoccupied, dismissive, and fearful attachment styles. The Spanish version has demonstrated high reliability [55].

#### Social Support

Measured with the Medical Outcomes Study-Social Support Survey (MOS-SSS) [56], analyzing perception of social support. The Spanish version [57] has good psychometric properties. Cronbach α was 0.96.

#### Quality of Compassion Meditation Practice

Assessed using the Compassion Practice Quality Questionnaire [58]. Cronbach α was 0.95.

### Acceptability Outcome Measures of Intervention

#### Expectations and Satisfaction

Measured using an adaptation of Borkovec and Nau’s [59] questionnaire by Campos et al [37]. This scale includes 8 items rated from 0 (“not at all”) to 4 (“very much”). The final score is obtained by adding the scores on each item. Scores range from 0 to 32, with higher scores representing higher expectations and satisfaction levels. Cronbach α was 0.95.

#### Usability

Assessed with an adaptation of the System Usability Scale by Campos et al [37]. This scale includes 10 statements rated on a 5-point scale measuring agreement with the statement (0=strongly disagree; 4=strongly agree). The final score is obtained by adding the scores on each item and multiplying the result by 2.5. Scores range from 0 to 100, where higher scores indicate better usability. Cronbach α was 0.90.

#### Participant Opinions

To explore reasons for dropout and nonparticipation, an online questionnaire was administered to participants who dropped out or declined to participate. The instrument included preset response options (multiple selections allowed) and open-ended text fields enabling participants to elaborate on their responses. A final set of 3 open-ended questions assessed general opinions about the intervention program. The instrument was adapted from previous studies [60,61].

### Harms

Adverse events and harms were monitored throughout the trial on a nonsystematic basis via passive surveillance. Participants were able to report any adverse experiences at any time through the study platform or the dedicated research team email address. No formal harms assessment instrument was used. The research team reviewed all participant communications throughout the trial period for any indication of psychological distress or unintended effects potentially attributable to the intervention. Data on harms were collected across the full trial period, from randomization to 6-month follow-up.

### Sample Size

Sample size was calculated using G*Power 3.1.9.7 (Heinrich Heine University Düsseldorf) [62]. Based on anticipated medium effect sizes (Cohen *d*=0.4), a statistical power of 0.80, and *α*=.05, the required sample was 52 participants for a repeated-measures design [63]. Accounting for an expected 30% dropout rate in internet-based interventions for chronic medical conditions [64-66], a minimum target of 34 participants per group was established, for a total minimum sample of 68 participants.

No interim analyses were planned or conducted during the trial. No formal stopping guidelines were prespecified.

### Randomization

#### Sequence Generation

Participants who met eligibility criteria were randomly allocated to either iABCT or WL control at a 1:1 ratio. Randomization was stratified by type of chronic medical condition (diabetes, fibromyalgia, intestinal inflammatory illness, migraines, low-back chronic pain, and other conditions). The allocation sequence was generated by an external researcher blind to the study using a computerized random generator (Random Allocation Software 2.0; Mahmood Saghaei). Randomization was performed using permuted blocks with randomly varied block sizes to prevent prediction of group assignment. Block sizes were not disclosed to the research team.

#### Allocation Concealment Mechanism

The randomization sequence was generated and held exclusively by the external researcher prior to participant enrollment. Research team members responsible for eligibility screening and enrollment did not have access to the allocation sequence at any point prior to assignment.

#### Implementation

After a participant completed the eligibility assessment and provided informed consent, their group assignment was communicated by the external researcher via the pregenerated randomization scheme. The personnel responsible for enrolling participants were therefore different from those generating and holding the allocation sequence.

#### Blinding

Blinding of participants and researchers was not possible due to the nature of the intervention, as participants were aware of their assigned condition. Data were analyzed by researchers who had access to group allocation.

### Statistical Methods

Data analyses were conducted using SPSS (version 28.0; IBM Corp) [67]. Baseline equivalence between groups was assessed using *t* tests, ANOVAs, and chi-square analyses. To address missing data and assess the effectiveness of the intervention, an intention-to-treat (ITT) approach was applied using linear mixed-effects models without using ad hoc imputation methods [43]. This analytic strategy is particularly suitable for RCTs involving repeated measures across multiple time points and is known for its robustness to violations of normality and other distributional assumptions [68,69].

The primary estimand was the time×condition interaction effect at 3-month follow-up for the 2 coprimary outcomes (EQ-5D and PHI). Total scores were prespecified as primary targets for all outcomes; subscale-level analyses were exploratory. Following the preregistered protocol, no alpha-adjustment for multiple primary outcomes was prespecified. EQ-5D and PHI were analyzed as coprimary end points without hierarchical testing or Bonferroni correction.

Separate mixed-effects models were conducted for each outcome variable using the MIXED procedure with REML estimation. Fixed effects included time, condition, and the time×condition interaction. Models incorporated a random intercept for each participant with an identity covariance structure. Denominator degrees of freedom for *F* tests were calculated using the Satterthwaite approximation. The variable “time” (baseline, 3-month follow-up, and 6-month follow-up) was modeled as a within-subject factor, while the experimental condition (iABCT vs WL) served as a between-subject factor. Primary efficacy analyses tested between-group differences at the 3-month follow-up (time×condition interaction). Six-month data were analyzed as within-group comparisons (baseline to 6-month) separately for each condition to assess (1) maintenance of treatment effects in the iABCT group and (2) postintervention changes in the WL group following delayed intervention access. Where statistically significant effects were observed, follow-up pairwise comparisons were performed using Bonferroni corrections. Effect sizes (Cohen *d*) and corresponding 95% CIs were computed to quantify both within- and between-group differences using standardized mean differences based on observed means and SDs. A small-sample bias correction factor *c(m*) was applied following Hedges and Olkin [70], yielding values equivalent to Hedges’ *g*; given correction factors ranging from 0.98 to 0.99 across analyses, differences from uncorrected Cohen *d* are negligible [71,72].

For qualitative data, participants’ responses regarding barriers to study enrollment (nonparticipants) and barriers to intervention engagement (dropouts) were explored using qualitative content analysis with a coding and categorization approach, including word frequency counts with ATLAS.ti software (v.23; ATLAS.ti Scientific Software Development GmbH). One researcher (MM-S) conducted initial open coding to identify facilitators and barriers. Codes were then reviewed by a second researcher (MDV) with experience in qualitative analysis. Discrepancies were discussed until consensus was reached. Additionally, thematic analysis following Braun and Clarke’s [73] methodology was conducted to identify common themes and patterns related to participant opinions on different aspects of the intervention program. Main findings from both analyses are presented in the “Results” section, while complete qualitative analysis including all tables, participant quotes, and detailed statistical breakdowns are provided in Multimedia Appendix 1.

### Ethical Considerations

This trial was conducted in compliance with the study protocol [38], the Declaration of Helsinki, and good clinical practice. Ethical approval for this trial was obtained from the Ethics Committee for Human Research of the University of Valencia (UV-INV_ETICA-1564960). The trial was prospectively registered in March 2021, prior to the enrollment of the first participant (study start: July 8, 2021).

Informed consent was obtained online before any study procedures. The online consent form included details of the trial, an explanation of potential risks and benefits, and contact information for the research team. Participants were informed that participation was completely optional and could be discontinued at any time.

Participants did not receive any financial compensation for their participation. Access to the intervention was provided free of charge to all participants.

All study data were collected and stored anonymously and deidentified. Participants were assigned unique identification codes, and no personally identifiable information was linked to study responses. Data were stored on secure servers with restricted access limited to the research team. This study did not involve the collection of images, photographs, or any visual material that could identify individual participants. All figures and tables included in the paper and Multimedia Appendix 1 contain only aggregated or anonymized data, and no identification of individual participants is possible.

## Results

### Participants Flow

**Figure 1.**
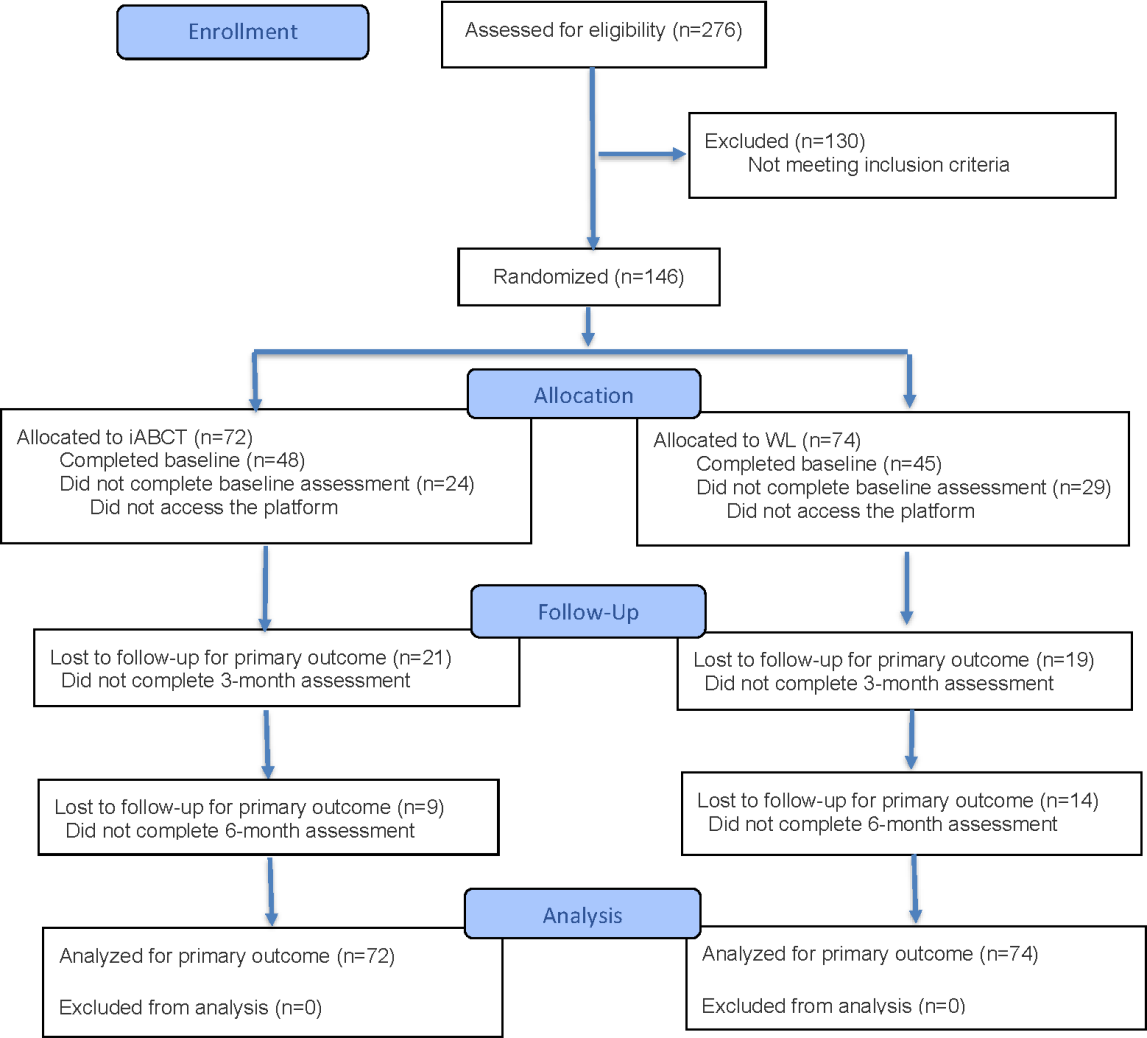
Participant flow through the 2-arm parallel randomized controlled trial evaluating internet-delivered attachment-based compassion therapy versus waiting list in adults with chronic medical conditions, from recruitment to 6-month follow-up. iABCT: internet attachment-based compassion therapy; WL: waiting list.

Initially, 276 participants were interested in the study, and 130 were excluded because they did not fulfill eligibility criteria (Figure 1). Thus, 146 participants were randomized (iABCT=72; WL=74). Of these, 53 participants did not access the platform (iABCT=24; WL=29), resulting in 93 participants who completed the baseline assessment (iABCT=48; WL=45). Little MCAR test confirmed that missing data were completely at random (*χ*²_153_=150.97, *P*=.53).

Dropout rates from baseline to 3-month follow-up were 43.75% for the iABCT group (21/48) and 42.22% for the WL group (19/45), resulting in 27 and 26 participants, respectively. Between 3-month and 6-month follow-up, additional dropout rates were 33.33% (9/27) for the iABCT group and 53.85% (14/26) for the WL group, with 18 and 12 participants completing the final assessment, respectively.

### Recruitment

Recruitment took place from April 2021 to April 2022. The last participant completed the 6-month follow-up assessment in October 2022. Each participant’s total follow-up duration was 6 months from randomization.

### Intervention and Comparator Delivery

The iABCT intervention was fully self-administered via the online platform, with no human therapist involved in delivery. Of the 72 participants randomized to iABCT, 24 (33.3%) did not access the platform and therefore received no intervention content. Systematic data on individual module completion were not collected. Adherence was monitored indirectly through platform login records, which triggered automated email reminders when participants had not logged in for 1 week. For the WL group, 29 of 74 (39.2%) randomized participants did not access the platform following delayed intervention access.

### Baseline Data

Table 2 shows participants’ sociodemographic data for each condition. Groups were comparable on all demographic variables.

**Table 2.** Sociodemographic characteristics of Spanish-speaking adults with chronic medical conditions randomized to internet attachment-based compassion therapy or waiting list in a 2-arm parallel randomized controlled trial at baseline^a^.

Characteristic	iABCT^b^ (N=72)	WL^c^ (N=74)
Age (years), mean (SD)	48.18 (11.00)	47.39 (11.14)
Sex, n (%)		
Male	13 (18.10)	9 (12.2)
Female	59 (81.90)	65 (87.8)
Chronic condition, n (%)		
Fibromyalgia	15 (20.8)	14 (18.9)
Colon disease	21 (29.2)	20 (27)
Diabetes	8 (11.1)	7 (9.5)
Migraine	2 (2.8)	4 (5.4)
Other	26 (36.1)	29 (39.2)
Comorbidity, n (%)	27 (37.50)	23 (31.1)
Marital status, n (%)		
Single	12 (16.70)	18 (24.30)
Married or in a relationship	51 (70.80)	47 (63.50)
Separated or divorced	9 (12.50)	8 (10.80)
Widowed	0 (0.00)	1 (1.40)
Educational level, n (%)		
Primary studies	8 (11.10)	11 (14.90)
Secondary studies	23 (31.90)	19 (25.70)
University studies	41 (56.90)	43 (58.10)
Others	0 (0.00)	1 (1.40)
Occupation, n (%)		
Unemployed	12 (16.70)	10 (13.50)
Student	3 (4.20)	3 (4.10)
Household work	1 (1.40)	0 (0.00)
Employed	38 (52.80)	43 (58.10)
Time off work	4 (5.60)	4 (5.40)
Retired	7 (9.70)	3 (4.10)
Permanent disability	6 (8.30)	7 (9.50)
Others	1 (1.40)	4 (5.40)

a A detailed breakdown of the conditions included in the “Other” category by group is provided in Table S2 in Multimedia Appendix 1.

b iABCT: internet attachment-based compassion therapy.

c WL: waiting list.

Regarding meditation experience, 37.7% (55/146) of participants had previous meditation experience, with similar proportions in both groups. Among experienced meditators, groups showed comparable frequency, duration, lifetime practice, and context of practice. Complete meditation experience data are provided in Table S3 in Multimedia Appendix 1.

Regarding clinical outcomes (Table 3), groups showed comparable scores on most measures. Descriptively, the WL group had higher self-compassion scores, while the iABCT group showed numerically higher self-criticism. All other outcomes showed similar baseline values across groups.

**Table 3.** Baseline clinical characteristics of adults with chronic medical conditions randomized to internet attachment-based compassion therapy or waiting list in a 2-arm parallel randomized controlled trial.

Measure	iABCT^a^ (n=48), mean (SD)	WL^b^ (n=45), mean (SD)
Quality of life		
EQ-5D		
Overall quality of life	–0.10 (0.78)	0.02 (0.75)
General health	52.29 (17.41)	58.00 (19.61)
Well-being		
PHI^c^		
Total well-being	5.91 (1.99)	6.32 (1.72)
Remembered well-being	5.92 (2.03)	6.36 (1.75)
Experienced well-being	5.76 (2.46)	5.87 (2.26)
Compassion and self-compassion		
SOCS-S^d^		
SOCS total score	66.78 (11.60)	73.67 (11.81)
Self-care behaviors		
B-MSCS^e^		
Physical care	2.79 (0.55)	2.84 (0.52)
Mindful relaxation	2.27 (0.94)	2.58 (0.92)
Self-compassion and purpose	2.63 (1.06)	3.06 (1.15)
Supportive relationships	3.74 (1.05)	3.59 (1.03)
Supportive structure	3.16 (0.92)	3.35 (1.04)
Mindfulness	3.29 (1.15)	3.66 (1.06)
Self-criticism		
SCRS^f^		
SCRS total score	26.76 (7.68)	23.40 (8.12)
Symptomatology		
DASS-21^g^		
Anxiety	13.32 (11.13)	12.48 (10.39)
Depression	18.14 (12.44)	16.05 (12.26)
Stress	21.14 (10.22)	20.19 (10.42)
DASS total score	52.59 (31.43)	48.71 (30.41)
Attachment styles		
RQ^h^		
Secure attachment	4.56 (2.05)	4.86 (1.89)
Preoccupied attachment	3.70 (2.21)	3.19 (2.10)
Dismissive attachment	3.95 (1.80)	4.24 (1.75)
Fearful attachment	3.81 (2.20)	3.17 (2.13)
Social support		
MOS-SSS^i^		
MOS-SSS total score	74.60 (17.05)	73.67 (15.31)

a iABCT: internet attachment-based compassion therapy.

b WL: waiting list.

c PHI: Pemberton Happiness Index.

d SOCS-S: Sussex-Oxford Compassion for the Self Scale.

e B-MSCS: Mindful Self-Care Scale - Brief version.

f SCRS: Self-Critical Rumination Scale.

g DASS-21: Depression, Anxiety, and Stress Scale.

h RQ: Relationships Questionnaire.

i MOS-SSS: Medical Outcomes Study-Social Support Survey.

### Numbers Analyzed, Outcomes, and Estimation

#### Results in Primary Outcomes at 3-Month Follow-Up

Results for primary outcomes at 3-month follow-up are presented in Table 4 (between-group effects). Complete descriptive statistics and within-group effect sizes are available in Table S4 in Multimedia Appendix 1.

Regarding quality of life, mixed-effects models showed a significant time×condition interaction for overall quality of life (*F*_1, 68.01_=6.33; *P*=.01). Between-group comparisons at 3-month follow-up revealed that participants in the iABCT condition scored significantly higher than the WL group, with a medium effect size (Cohen *d*=0.62, 95% CI 0.07-1.17). Within-group analyses showed significant improvements in the iABCT group for overall quality of life (Cohen *d*=0.60, 95% CI 0.30-0.89) and general health (Cohen *d*=0.61, 95% CI 0.37-0.84), whereas no significant changes were observed in the WL group.

**Table 4.** Between-group mean differences and effect sizes at 3-month follow-up for primary outcomes (quality of life and well-being) in Spanish-speaking adults with chronic medical conditions randomized to internet attachment-based compassion therapy versus waiting list in a 2-arm parallel randomized controlled trial.

Measure	Condition	3-month follow-up	
		Mean difference (iABCT^a^ vs WL^b^)	Between-group effect size, Cohen *d* (95% CI)
Quality of life			
EQ-5D			
Overall quality of life	iABCT WL	0.46^c^	0.62 (0.07 to 1.17)
General health	iABCT WL	0.32	0.05 (−0.49 to 0.58)
Well-being			
PHI^d^			
Total well-being	iABCT WL	0.48	0.21 (−0.33 to 0.75)
Remembered well-being	iABCT WL	0.49	0.21 (−0.33 to 0.75)
Experienced well-being	iABCT WL	0.35	0.13 (−0.41 to 0.67)

a iABCT: internet attachment-based compassion therapy.

b WL: waiting list.

c *P*<.05.

d PHI: Pemberton Happiness Index.

In relation to well-being, results showed a significant condition×time interaction effect on total well-being (*F*_1, 60.72_=4.69; *P*=.03) and remembered well-being (*F*_1, 61.79_=4.71; *P*=.03). However, between-group comparisons at 3-month follow-up were not statistically significant (total well-being: Cohen *d*=0.21, 95% CI −0.33 to 0.75; remembered well-being: Cohen *d*=0.21, 95% CI −0.33 to 0.75; refer to Table 4). Within-group analyses showed significant increases at 3-month follow-up in total well-being (Cohen *d*=0.26, 95% CI 0.03-0.48) and remembered well-being (Cohen *d*=0.27, 95% CI 0.04-0.50), but no significant change was found in the WL group. Although between-group differences did not reach statistical significance, participants in the iABCT condition scored numerically higher on well-being than the WL group.

#### Results in Secondary Outcomes at 3-Month Follow-Up

##### Overview

Results for secondary outcomes at 3-month follow-up are presented in Table 5 (between-group effects). Complete descriptive statistics and within-group effect sizes are available in Table S5 in Multimedia Appendix 1.

**Table 5.** Between-group mean differences and effect sizes at 3-month follow-up for secondary outcomes in Spanish-speaking adults with chronic medical conditions randomized to internet attachment-based compassion therapy versus waiting list in a 2-arm parallel randomized controlled trial.

Measure	Condition	3-month follow-up	
		Mean difference (iABCT^a^ vs WL^b^)	Between-group effect size, Cohen *d* (95% CI)
Compassion and self-compassion			
SOCS-S^c^			
SOCS total score	iABCT WL	−0.65	0.07 (−0.47 to 0.62)
Self-care behaviors			
B-MSCS^d^			
Physical care	iABCT WL	−0.10	−0.19 (−0.73 to 0.35)
Mindful relaxation	iABCT WL	−0.36	−0.33 (−0.88 to 0.21)
Self-compassion and purpose	iABCT WL	0.17	0.17 (−0.37 to 0.71)
Supportive relationships	iABCT WL	0.43	0.49 (−0.06 to 1.03)
Supportive structure	iABCT WL	0.03	0.18 (−0.36 to 0.72)
Mindfulness	iABCT WL	−0.03	0.02 (−0.52 to 0.56)
Self-criticism			
SCRS^e^			
SCRS total score	iABCT WL	−1.45	−0.26 (−0.82 to 0.29)
Symptomatology			
DASS-21^f^			
Anxiety	iABCT WL	0.07	−0.03 (−0.58 to 0.52)
Depression	iABCT WL	0.42	0.08 (−0.48 to 0.63)
Stress	iABCT WL	−0.49	−0.09 (−0.64 to 0.46)
DASS total score	iABCT WL	0.42	−0.02 (−0.58 to 0.54)
Attachment styles			
RQ^g^			
Secure attachment	iABCT WL	1.07	0.47 (−0.09 to 1.02)
Preoccupied attachment	iABCT WL	−1.28^h^	−0.59 (−1.15 to −0.03)
Dismissive attachment	iABCT WL	−0.11	−0.09 (−0.64 to 0.46)
Fearful attachment	iABCT WL	−0.36	−0.12 (−0.67 to 0.43)
Social support			
MOS-SSS^i^			
MOS-SSS total score	iABCT	6.16	0.32 (−0.24 to 0.88)

a iABCT: internet attachment-based compassion therapy.

b WL: waiting list.

c SOCS-S: Sussex-Oxford Compassion for the Self Scale.

d B-MSCS: Mindful Self-Care Scale - Brief version.

e SCRS: Self-Critical Rumination Scale.

f DASS-21: Depression, Anxiety, and Stress Scale.

g RQ: Relationships Questionnaire.

h *P*<.05.

i MOS-SSS: Medical Outcomes Study-Social Support Survey.

##### Compassion and Self-Compassion (SOCS-S Scores)

Mixed-effects models showed a significant time×condition interaction (*F*_1, 59.95_=4.90; *P*=.03). However, between-group comparisons at 3-month follow-up did not reveal statistically significant differences (Cohen *d*=0.07, 95% CI −0.47 to 0.62; Table 5). Within-group comparisons showed significant increases in the iABCT group (Cohen *d*=0.66, 95% CI 0.38-0.94), but no significant changes in the WL group (Table S4 in Multimedia Appendix 1).

##### Self-Care Behaviors (B-MSCS Scores)

No significant time×condition interactions were found for any B-MSCS subscale. Between-group comparisons at 3-month follow-up did not reveal statistically significant differences for any domain (Table 5). Within-group analyses indicated that the iABCT group showed increases in self-compassion and purpose (Cohen *d*=0.60, 95% CI 0.24-0.95) and supportive structure (Cohen *d*=0.39, 95% CI 0.13-0.64), while no significant changes were observed in the WL group (Table S4 in Multimedia Appendix 1).

##### Self-Criticism (SCRS Scores)

Mixed-effects models showed a significant time × condition interaction (*F*_1, 61.16_=6.76; *P*=.01). However, between-group comparisons at 3-month follow-up were not statistically significant (Cohen *d*=−0.26, 95% CI −0.82 to 0.29; Table 5). Within-group comparisons revealed significant reductions in the iABCT group (Cohen *d*=0.67, 95% CI 0.40-0.94), but no changes in the WL group (Table S4 in Multimedia Appendix 1).

##### Symptomatology (DASS-21 Scores)

No significant time×condition interactions were found for any DASS-21 subscale. Between-group comparisons at 3-month follow-up did not reveal statistically significant differences (Table 5). Within-group comparisons showed reductions in both groups: iABCT (Cohen *d*=0.57, 95% CI 0.32-0.82) and WL (Cohen *d*=0.45, 95% CI 0.22-0.67) for total DASS-21 scores, with low to medium effect sizes across anxiety, depression, and stress subscales.

##### Attachment Styles (RQ Scores)

Mixed-effects models showed significant time × condition interactions for secure attachment (*F*_1, 66.43_=7.15; *P*=.01) and preoccupied attachment *(F*_1, 65.53_=10.19; *P*=.002). Between-group comparisons at 3-month follow-up revealed significantly lower preoccupied attachment in the iABCT group (Cohen *d*=−0.59, 95% CI −1.15 to−0.03), while secure attachment did not reach statistical significance (Table 5). Within-group comparisons showed significant increases in secure attachment and reductions in preoccupied and fearful attachment in the iABCT group (Cohen *d*=0.25, 95% CI 0.02-0.48; Cohen *d*=0.55, 95% CI 0.26-0.83; and Cohen *d*=0.63, 95% CI 0.36-0.91, respectively). No significant changes were found in the WL group.

##### Social Support (MOSS-S Scores)

No significant time×condition interaction was found for MOS-SSS. Between-group comparisons at 3-month follow-up did not reveal statistically significant differences (Cohen *d*=0.32, 95% CI −0.24 to 0.88; Table 5). No significant within-group changes were observed in either condition.

### Efficacy of the Intervention at 6-Month Follow-Up

#### Maintenance of Changes at 6-Month Follow-Up for the iABCT Condition

Regarding results on primary measures, a significant effect of time was found from baseline to 6-month follow-up on overall quality of life (*F*_2, 52.83_=6.50; *P*=.003) and general health subscales of the EQ-5D (*F*_2, 56.41_=4.89; *P*=.01), but no significant changes were found for any subscale of the PHI. Within-group comparisons revealed significant pre- to 6-month follow-up increases in overall quality of life and general health corresponding to medium and low effect sizes (Cohen *d*=0.54, 95% CI 0.32-0.77; Cohen *d*=0.44, 95% CI 0.19-0.69, respectively; refer to Table 4).

Results on secondary measures revealed a significant effect of time at 6-month follow-up on compassion total score (*F*_2, 50.17_=7.61; *P*=.001), self-criticism total score (*F*_2, 51.50_=6.04; *P*=.004), overall symptomatology (*F*_2, 42.25_=10.59; *P*<.001) and preoccupied (*F*_2, 54.01_=7.35; *P*=.002), fearful (*F*_2, 53.86_=7.38; *P*=.001), and dismissive attachment (*F*_2, 53.91_=3.70; *P*=.03). Within-group comparison showed significant increases on compassion total score with a medium effect size (Cohen *d*=0.64, 95% CI 0.42-0.86) and significant pre- to-6-month follow-up reductions on self-criticism were shown with low effect sizes (Cohen *d*=0.47, 95% CI 0.28-0.66; refer to Table 5). Moreover, results showed significant reductions in all subscales of the DASS corresponding to low effect size for anxiety (Cohen *d*=0.34, 95% CI 0.16-0.52) and medium effect sizes for depression, stress, and overall symptomatology (Cohen *d*=0.50, 95% CI 0.28-0.72; Cohen *d*=0.67, 95% CI 0.42-0.92; and Cohen *d*=0.54, 95% CI 0.34-0.73, respectively). Significant pre- to-6-month reductions were also shown with medium and low effect sizes on dismissive attachment (Cohen *d*=.067, 95% CI 0.40-0.95), preoccupied attachment (Cohen *d*=0.68, 95% CI 0.49-0.97), and fearful attachment (Cohen *d*=0.44, 95% CI 0.20-0.68).

No significant pre- to 6-month follow-up changes were found on social support (MOSS-S scores).

#### The Effectiveness of the Intervention at 6-Month Follow-Up for the WL Group.

Regarding primary outcomes, results showed no significant changes from baseline to 6-month follow-up on quality of life (EQ-5D scores) or well-being (PHI scores) for the WL condition (refer to Table 4 for more details).

Results on secondary outcomes show significant effects of time from baseline to 6-month follow-up on the physical care subscale of the B-MSCS (*F*_2, 52.21_=3.25; *P*=.05) and the depression subscale of the DASS-21 (*F*_2, 39.96_=7.77; *P*=.001).

Within-group comparisons revealed significant pre- to 6-month follow-up changes in the WL group for the physical care subscale with a medium effect size (Cohen *d*=0.68, 95% CI 0.38-0.99) and a significant reduction in depression symptomatology corresponding to a medium effect size (Cohen *d*=0.42, 95% CI 0.19-0.66).

No significant changes were found in the WL group at 6-month follow-up for any other secondary outcome (Table 5).

#### Treatment Acceptability: Satisfaction and Usability

In response to questions about satisfaction with the program, most participants from the iABCT group showed high satisfaction with the program (mean 26.06, SD 5.74) after finishing it (at 3 months). Specifically, the majority of participants were satisfied with the quantity (mean 3.47, SD 0.72) and quality (mean 3.41*,* SD 0.71) of the services received, and they rated the program as helpful for solving their problems (mean 2.35, SD 0.93) and for dealing better with their problems (mean 3.29, SD 0.77), and they considered that the help received was what they expected (mean 3.24, SD 0.97). Moreover, the participants’ overall satisfaction with the program was high (mean 3.53, SD 0.80), and most participants would use the program again in case they needed it (mean 3.35, SD 0.93), and they would recommend it to a friend (mean 3.41, SD 0.80).

Results of usability show that participants from the iABCT group rated the program as functional and easy to use in overall terms (mean 82.79, SD 18.64) on a 0‐100 scale, indicating excellent usability (scores>80). These results indicate that overall participants had a positive experience in terms of the program usability and acceptance of technology.

Regarding results on difficulties of compassion-based meditation, most participants show a high quality of compassion practice, referring to fewer difficulties in different key aspects of compassion practice, such as mental imagery or a sense of connection and warmth (mean 61.65, SD 24.34).

Regarding the WL condition after giving access to the intervention and finishing it (at 6 months), most participants show high satisfaction with the program (mean 24.14, SD 2.19). Most participants were satisfied with the quantity (mean 2.86, SD 0.38) and quality (mean 3.43, SD 0.53) of services received, and they considered that the help received was what they expected (mean 3.00, SD 0.00). Participants from the WL condition rated the program as useful for solving their problems (mean 2.14, SD 0.38) and helpful for dealing better with their problems (mean 3.29, SD 0.49). Moreover, participants’ overall satisfaction with the program was high (mean 3.14, SD 0.38), and most participants would use the program again in case they needed it (mean 3.14, SD 0.69), and they would recommend it to a friend dealing with similar difficulties (mean 3.14, SD 0.69).

Similarly, participants from the WL condition rated results regarding usability, showing that participants from the WL condition rated the program as easy to use (mean 78.33, SD 21.83), which indicates that they found the program functional.

Finally, regarding the quality of compassion meditation practice, some difficulties were found by participants in the WL condition regarding aspects of the compassion meditation practice, such as mental imagery, sense of connection and warmth, or compassionate phrases and gestures (mean 49.71, SD 30.12).

### Implementation Barriers and Facilitators

#### Overview

A total of 74 participants who dropped out of the study or decided not to participate were contacted to complete the online questionnaire assessing reasons for nonengagement and general opinions about the intervention program. Responses were completed by 34 participants (46% response rate): 21 from the nonparticipation group and 13 from the dropout group.

#### Implementation Barriers

Two distinct types of barriers were identified (Tables S4 and S5 in Multimedia Appendix 1). Barriers to study enrollment (reported by 21 nonparticipants who declined participation after initial contact) included lack of motivation (19/21, 90.5%), excessive access steps (14/21, 66.7%), and limited interest in the program (13/21, 61.9%). Barriers to intervention engagement (reported by 13 participants who dropped out after baseline) included need for contact with a physical therapist (12/13, 92.3%), insufficient monitoring and support (8/13, 61.5%), and program demands exceeding expectations (8/13, 61.5%).

#### Facilitators and Desired Improvements

Participants consistently identified desired improvements, including increased therapist presence (27/34, 79.4%), more attractive and user-friendly design (25/34, 73.5%), enhanced interactivity (15/34, 55.9%), and shorter program duration (15/34, 55.9%). Other frequently mentioned improvements included more tailored and specific programming (11/34, 32.4%) and functionalities to interact with others (11/34, 32.4%; Table S8 in Multimedia Appendix 1).

#### Thematic Analysis

Thematic analysis revealed 4 main domains of feedback (Table S9 in Multimedia Appendix 1): support and interaction (29.75% of comments), program characteristics (26.45%), initial assessment and access aspects (14.88%), and web platform features (28.93%). Most participants emphasized the need for increased human support, particularly from therapists, and program modifications to enhance engagement and usability.

### Harms

No deaths occurred during the trial. No participants withdrew from the study due to adverse events or harms. No serious adverse events were identified through passive surveillance over the full trial period (randomization to 6-month follow-up) in either the iABCT or WL group.

### Ancillary Analyses

No subgroup analyses were prespecified or conducted in this trial. Sensitivity analyses using multiple imputation methods were prespecified in the registered protocol but were not conducted (see the “Changes to Trial Protocol” section). All other analyses reported were prespecified in the registered protocol, except for the exploratory subscale-level analyses, which were post hoc.

## Discussion

### Principal Findings

#### Overview

This RCT examined the efficacy of a fully self-guided, iABCT for adults with chronic medical conditions, compared to a WL control. The first hypothesis was supported for quality of life, which showed significant between-group improvements at 3-month follow-up, but was not supported for well-being, which showed improvements in the expected direction that did not reach statistical significance. The second hypothesis was supported for preoccupied attachment, which showed significant between-group reductions in the iABCT condition compared to the WL, but was not supported for the remaining secondary outcomes. Self-compassion, self-criticism, psychological symptoms, and social support showed within-group improvements without reaching statistical significance in between-group comparisons. Quality of life improvements were maintained within the iABCT group at 6-month follow-up, alongside sustained improvements in self-compassion, self-criticism, psychological symptoms, and attachment styles. The third hypothesis was supported: participants reported high satisfaction and usability, though qualitative analyses revealed important engagement barriers related to the fully self-guided format. No serious adverse events were identified in either group, suggesting an acceptable safety profile consistent with prior CBI research [74].

#### Efficacy of iABCT: Primary Outcomes

The significant between-group improvement in overall quality of life favoring iABCT at 3-month follow-up suggests that the intervention may effectively address the subjective burden of chronic illness through theoretically proposed mechanisms, including enhanced acceptance of suffering, promotion of adaptive coping strategies such as positive reframing and self-care behaviors, and reduction of maladaptive patterns such as self-blame [26,36]. Meta-analytic evidence supports that self-compassion interventions in chronic illness populations reduce psychological distress through these pathways, which may in turn improve health-related quality of life [22,24].

These findings align with prior evidence supporting the effectiveness of CBIs for quality of life in chronic medical populations. Notably, face-to-face ABCT has shown large effect sizes in patients with fibromyalgia [30], and systematic reviews consistently demonstrate benefits of CBIs across diverse chronic conditions [24,75]. The present results extend this evidence to fully self-guided, internet-delivered formats, supporting digital CBIs as potentially viable alternatives to face-to-face delivery [76-78]. However, the absence of preplanned alpha adjustment for dual primary outcomes increases the possibility of type I error, and these findings should therefore be interpreted cautiously as exploratory, pending replication in adequately powered confirmatory trials.

The absence of significant between-group differences in well-being, despite within-group improvements in the iABCT condition, warrants consideration. Well-being may be more sensitive to fluctuations in physical health status than quality of life, making sustained gains particularly challenging in chronic illness populations where comorbidity rates are high and functional limitations may progress over time [79]. Additionally, quality of life improvements were maintained within the iABCT group at 6-month follow-up with medium effect sizes, whereas well-being gains were not sustained. This pattern suggests that ongoing or booster support beyond the initial intervention period may be needed to maintain psychological gains, particularly for well-being outcomes [79]. Interpretation of long-term effects is further limited by high attrition at 6-month follow-up in both groups, which is consistent with evidence that dropout rates increase with longer follow-up periods in RCTs [80].

#### Efficacy of iABCT: Secondary Outcomes

The significant between-group reduction in preoccupied attachment in the iABCT condition is consistent with theoretical links between compassion cultivation, soothing system activation, and secure attachment orientation [81-84]. This finding extends previous evidence from face-to-face ABCT in healthy populations to chronic illness samples using fully self-guided digital formats. The absence of significant between-group effects for most other secondary outcomes, including self-compassion, self-criticism, self-care behaviors, psychological symptoms, and social support, limits conclusions about intervention-specific mechanisms. Notably, both the iABCT and waiting list groups showed reductions in psychological symptoms over time, which is consistent with evidence of spontaneous symptom reduction, regression to the mean, or nonspecific effects of study participation in waitlist control designs [85-87]. While within-group improvements in self-compassion and self-criticism align with patterns observed in other CBIs [74,88-91], and theoretical models link self-compassion with self-care behaviors and social connectedness [92-94], this study was likely underpowered to detect modest between-group differences given the high attrition rates observed [80]. Future adequately powered trials are therefore needed to determine whether these within-group patterns reflect true intervention-specific effects or study artifacts.

At 6-month follow-up, improvements in self-compassion, self-criticism, psychological symptoms, and attachment styles were maintained within the iABCT group with low to medium effect sizes, while self-care behaviors and social support showed no significant long-term changes. The absence of sustained gains in self-care behaviors is noteworthy, as self-care is a key target of CBIs in chronic illness management [25,26]. This may reflect the need for more sustained practice and support beyond the 8-week intervention period or may indicate that self-care behavioral change requires additional tailoring to the specific demands of different chronic conditions [95].

#### Treatment Acceptability and Engagement Barriers

The high levels of satisfaction and usability reported by participants support the acceptability of iABCT for chronic illness populations and the feasibility of online delivery formats in this context [65,75,96,97]. Participants found the program functional and easy to use, and reported high quality of compassion meditation practice, which is consistent with evidence supporting the acceptability of CBIs across diverse chronic conditions [65].

Despite high satisfaction, qualitative analyses revealed critical engagement barriers that likely contributed to the observed attrition rates. Nonparticipation was primarily attributed to low motivation, excessive access steps, and limited program interest, while dropout after baseline was mainly related to the need for human therapist contact, insufficient monitoring and support, and program demands exceeding expectations. Participants consistently identified increased therapist presence, more attractive and user-friendly design, and enhanced interactivity as desired improvements. These patterns align with best practices emphasizing human support, personalization, and user-centered design in digital health interventions [32,98-101], and are consistent with broader evidence showing that guided internet-based interventions generally outperform unguided formats in terms of engagement and outcomes [98,99,101]. The observed attrition rate, while high, falls within the range reported for unguided digital interventions in chronic illness populations [65].

These qualitative findings should be interpreted with caution. Only around half of contacted individuals completed the assessment, potentially introducing response bias. Additionally, formal interrater reliability was not calculated, and thematic saturation was not prospectively assessed. These methodological considerations limit the generalizability of the qualitative findings, which should therefore be considered exploratory. Furthermore, elements inherent to the RCT context, such as automated email reminders and the availability of a dedicated research team contact, may have provided implicit support unlikely to be present in routine implementation, potentially inflating engagement rates relative to real-world deployment.

### Limitations

Several limitations warrant consideration. First, the waitlist control design rather than an active control condition may have contributed to effect overestimation, as waitlist controls have been shown to function as nocebo conditions in psychotherapy trials [85,86]. Second, high attrition rates, particularly at 6-month follow-up, may have compromised statistical power and the interpretability of long-term effects. Third, the identity covariance structure used in the mixed models, while necessary to ensure model convergence given the modest sample size and high attrition, may not adequately capture residual correlations between repeated measures. Misspecification of the covariance structure can bias SEs and affect statistical inference, particularly under the MAR assumption [102,103]. Fourth, the transdiagnostic approach, while beneficial for addressing comorbid conditions, prevented illness-specific analyses and may have limited intervention tailoring [104]. The heterogeneity of the sample and the unbalanced number of participants per chronic condition also prevented analysis of illness interference as a moderator, which has been shown to be relevant in CBIs [29]. Fifth, no multiplicity adjustment was prespecified for the dual primary outcomes or the multiple secondary outcomes, increasing the risk of type I error. Relatedly, between-group comparisons should be prioritized as the most rigorous test of intervention efficacy, and within-group improvements without corresponding between-group differences should be considered exploratory. Sixth, cost-effectiveness was not evaluated, limiting health care implementation assessments despite the considerable socioeconomic burden of chronic diseases [1,105]. Seventh, the absence of between-group comparisons at 6-month follow-up due to ethical considerations restricts conclusions about long-term maintenance of effects. Regarding the qualitative analyses, only around half of contacted individuals completed the questionnaire, formal interrater reliability was not calculated, and thematic saturation was not prospectively assessed, which limits the generalizability of those findings. Finally, the predominantly female, Spanish-speaking, and highly educated sample limits the generalizability of findings to broader chronic illness populations.

### Conclusions

This preliminary trial provides initial evidence that a fully self-guided, iABCT may improve overall quality of life in adults with chronic medical conditions, with effects maintained at 6-month follow-up. Significant reductions in preoccupied attachment were also observed. However, given the absence of alpha adjustment for dual primary outcomes, the high attrition rates, the modest sample size, and the waitlist control design, these findings should be interpreted cautiously as exploratory and require replication in adequately powered confirmatory trials with active control conditions and appropriate multiplicity correction. Improvements in both the iABCT and waitlist groups for some outcomes suggest potential contributions from assessment effects, natural symptom fluctuation, or regression to the mean, which further limits causal attribution. Despite high treatment satisfaction and acceptability, qualitative analyses revealed critical engagement barriers emphasizing the need for human support, personalized content, and user-centered design in digital CBIs.

This study makes several novel contributions to the field. To our knowledge, it provides the first empirical evidence from an RCT that a fully self-guided, internet-delivered attachment-based compassion intervention may produce improvements in quality of life across diverse chronic medical conditions, extending ABCT beyond its original face-to-face [29,30] and single-diagnosis [29] formats. Unlike existing digital compassion programs, iABCT uniquely integrates attachment security as a theoretical change mechanism alongside self-compassion cultivation, offering a theoretically grounded framework that may be particularly relevant for chronic illness populations whose psychological difficulties are often rooted in early relational experiences. Importantly, by targeting transdiagnostic psychological processes common across chronic medical conditions, iABCT is positioned as a potentially universal intervention capable of addressing shared mechanisms of suffering across the full spectrum of chronic illness. The scalable format offers potential to address unmet psychological needs in populations facing geographic, economic, or mobility-related access barriers [76-78], with promise for reducing health care burden [1,105]. Future research should prioritize adequately powered confirmatory trials with active control conditions, hierarchical testing procedures for multiple outcomes, and alternative covariance structures to ensure valid inference. Incorporating human support elements such as therapist guidance or peer support, developing condition-specific adaptations, investigating mechanisms of change, and conducting cost-effectiveness analyses are also key priorities to optimize both the reach and effectiveness of digital CBIs in real-world health care settings [32,98,106].

## Supplementary material

10.2196/86679 Multimedia Appendix 1 Descriptive statistics, effect sizes, and qualitative findings supporting the study results.

10.2196/86679 Checklist 1 CONSORT-EHEALTH V1.6.

## Abbreviations

ABCT: attachment-based compassion therapy
B-MSCS: Mindful Self-Care Scale - Brief version
CBI: compassion-based intervention
CONSORT: Consolidated Standards of Reporting Trials
DASS-21: Depression, Anxiety, and Stress Scale
iABCT: internet attachment-based compassion therapy
ICT: information and communications technology
ITT: intention-to-treat
MAR: missing-at-random
MOS-SSS: Medical Outcomes Study-Social Support Survey
PHI: Pemberton Happiness Index
RCT: randomized controlled trial
REML: Restricted Maximum Likelihood
RQ: Relationships Questionnaire
SCRS: Self-Critical Rumination Scale
SOCS-S: Sussex-Oxford Compassion for the Self Scale
SPIRIT: Standard Protocol Items: Recommendations for Interventional Trials
WL: waiting list
