# Supplementary material for: Internet Attachment–Based Compassion Therapy for Adults With Chronic Medical Conditions: Randomized Controlled Trial
Source: J Med Internet Res. 2026 Aug 11;28:e86679. doi: 10.2196/86679 (PMC13460065; doi:10.2196/86679)
Supplement: Supplementary file 2 [file jmir-v28-e86679-s001.docx]

**MULTIMEDIA APPENDIX 1**

**Table S1.** Descriptive statistics for disease-specific scales by condition at Pre- and Post- intervention.

|  |  | **WL** | | | | **iABCT** | | | |
| --- | --- | --- | --- | --- | --- | --- | --- | --- | --- |
|  |  | **Pre** | | **Post** | | **Pre** | | **Post** | |
| **Scale** | **Subscale** | ***M (SD)*** | ***n*** | ***M (SD)*** | ***n*** | ***M (SD)*** | ***n*** | ***M (SD)*** | ***n*** |
| **Diabetes Distress Scale (DDS)** | Total | 3.25 (0.93) | 5 | 2.92 (1.68) | 3 | 2.58 (1.26) | 8 | 1.85 (1.13) | 5 |
|  | Emotional Burden | 3.92 (1.13) | 5 | 3.00 (1.51) | 3 | 3.05 (1.31) | 8 | 2.20 (1.69) | 5 |
|  | Physician-related Distress | 2.60 (1.53) | 5 | 2.83 (1.63) | 3 | 2.22 (1.80) | 8 | 1.50 (0.61) | 5 |
|  | Regimen-related Distress | 3.04 (0.89) | 5 | 2.73 (1.72) | 3 | 2.70 (1.77) | 8 | 2.00 (1.47) | 5 |
|  | Interpersonal Distress | 3.33 (1.62) | 5 | 3.22 (2.04) | 3 | 2.08 (1.16) | 8 | 1.47 (1.04) | 5 |
| **Roland-Morris Questionnaire (RMQ)** | Total | — | — | — | — | 9.00 (NA) | 1 | — | — |
| **Revised Fibromyalgia Impact Questionnaire (FIQR)** | Total | 72.92 (6.91) | 6 | 63.37 (14.84) | 5 | 56.43 (9.04) | 9 | 51.79 (21.12) | 4 |
|  | Function | 21.83 (2.66) | 6 | 19.47 (8.35) | 5 | 14.48 (4.54) | 9 | 16.92 (6.62) | 4 |
|  | Overall Impact | 14.50 (2.95) | 6 | 13.60 (4.28) | 5 | 12.67 (3.57) | 9 | 8.00 (6.32) | 4 |
|  | Symptoms | 36.58 (3.02) | 6 | 30.30 (4.84) | 5 | 29.28 (3.96) | 9 | 26.88 (9.71) | 4 |
| **Migraine Disability Assessment (MIDAS)** | Total | — | — | 39.00 (NA) | 1 | — | — | — | — |
| **Inflammatory Bowel Disease Questionnaire (IBDQ)** | Total | 152.27 (31.19) | 11 | 141.67 (37.07) | 3 | 166.25 (30.22) | 12 | 167.50 (11.15) | 6 |
|  | Bowel Symptoms | 48.45 (11.23) | 11 | 43.33 (9.07) | 3 | 53.58 (11.08) | 12 | 53.17 (3.54) | 6 |
|  | Systemic Symptoms | 22.00 (6.07) | 11 | 19.67 (6.81) | 3 | 23.33 (5.93) | 12 | 20.33 (2.34) | 6 |
|  | Emotional Function | 55.82 (11.69) | 11 | 48.33 (15.50) | 3 | 58.17 (13.07) | 12 | 61.33 (6.38) | 6 |
|  | Social Function | 26.00 (7.90) | 11 | 30.33 (7.23) | 3 | 31.17 (3.83) | 12 | 32.67 (1.86) | 6 |
| **Illness Perception Questionnaire-Revised (IPQ-R)** | Control | 31.90 (4.98) | 40 | 31.73 (5.58) | 26 | 32.98 (3.77) | 43 | 32.96 (4.47) | 24 |
|  | Emotional Representations | 25.85 (5.25) | 40 | 26.62 (5.83) | 26 | 27.47 (6.21) | 43 | 26.96 (5.58) | 24 |
|  | Timeline (Acute/Chronic) | 16.07 (2.15) | 40 | 16.04 (1.97) | 26 | 16.19 (1.50) | 43 | 15.96 (1.60) | 24 |
|  | Timeline Cyclical | 15.05 (3.07) | 40 | 15.35 (3.72) | 26 | 14.30 (4.62) | 43 | 14.17 (3.76) | 23 |
|  | Illness Coherence | 9.12 (2.26) | 40 | 9.23 (2.73) | 26 | 8.56 (2.05) | 43 | 8.75 (2.09) | 24 |
|  | External Factors | 6.42 (2.41) | 40 | 6.96 (2.79) | 26 | 5.93 (2.47) | 43 | 5.91 (2.71) | 23 |
|  | Behaviour | 5.00 (2.49) | 40 | 5.62 (2.65) | 26 | 5.30 (2.35) | 43 | 6.30 (2.80) | 23 |
|  | Stress/Tension | 9.62 (4.03) | 40 | 9.77 (4.16) | 26 | 9.79 (3.32) | 43 | 10.88 (3.47) | 24 |
|  | Psychological Attributions | 8.65 (4.52) | 40 | 9.00 (4.91) | 26 | 9.93 (4.04) | 43 | 10.83 (3.87) | 24 |
|  | Heredity | 3.30 (1.45) | 40 | 3.15 (1.71) | 26 | 2.74 (1.47) | 43 | 2.88 (1.36) | 24 |

**Table S2.** Breakdown of chronic conditions within the ‘Other’ diagnostic category by group.

| **Condition** | **iABCT (*n* = 26)**  ***n (%)*** | **WL (*n* = 29)**  ***n (%)*** |
| --- | --- | --- |
| Autoimmune & inflammatory diseases | 8 (30.8%) | 11 (37.9%) |
| Musculoskeletal disorders | 5 (19.2%) | 2 (6.9%) |
| Chronic fatigue & sensitization syndromes | 4 (15.4%) | 4 (13.8%) |
| Respiratory diseases | 3 (11.5%) | 2 (6.9%) |
| Neurological conditions | 3 (11.5%) | 3 (10.3%) |
| Cardiovascular & metabolic conditions | 2 (7.7%) | 2 (6.9%) |
| Gynecological conditions | 0 (0.0%) | 2 (6.9%) |
| Other / unspecified | 1 (3.8%) | 3 (10.3%) |

*Note.* Percentages are based on the total 'Other' subgroup size for each condition (iABCT n = 26; WL = 29). iABCT: Internet Attachment-Based Compassion Therapy; WL = waiting list.

**Table S3.** Meditation practice data for each condition.

|  | **iABCT**  **n= 72** | **WL**  **n= 74** |
| --- | --- | --- |
| Meditation experience (%) | 34.70 | 40.50 |
| Frequency of meditation (%) |  |  |
| Daily | 8.00 | 14.30 |
| 3 or 4 times a week | 24.00 | 14.30 |
| Once a week or less | 20.00 | 21.40 |
| 2 or 3 times a month | 24.00 | 28.60 |
| Never | 24.00 | 21.40 |
| Duration of each session (minutes) M (SD) | 18.10 (12.79) | 17.44 (9.29) |
| Lifetime practice (years) M (SD) | 1.63 (1.34) | 1.37 (1.18) |
| Practice interruption (months)* M (SD) | 8.35 (10.95) | 11.85 (11.15) |
| Context of practice (%)* |  |  |
| Secular | 91.30 | 92.90 |
| Religious | 8.70 | 7.10 |

*Note: *only for experienced meditators*

**Table S4.** Means, standard deviations, and within-group effect sizes for primary outcomes assessed at baseline, 3-month, and 6-month follow-up in adults with chronic medical conditions randomized to Internet-Delivered Attachment-Based Compassion Therapy (iABCT) or waiting list (WL).

| **Measure** | **Condition** | **M (SD)** | | | **Within-group effect size, *d* [95% CI]** | | |
| --- | --- | --- | --- | --- | --- | --- | --- |
|  |  | **Pre** | **Post (3M)** | **FU (6M)** | **Pre vs. 3M** | | **Pre. Vs 6M** |
| **Quality of life**  *EQ-5D* |  |  |  |  |  |  | |
| Overall quality of life | iABCT | -0.10 (0.78) | 0.37 (0.74) | 0.33 (0.69) | **0.60 [0.30, 0.89]** | **0.54 [0.32, 0.77]** | |
|  | WL | 0.02 (0.75) | -0.08 (0.69) | 0.33 (0.78) | -0.13 [-0.17, 0.43] | 0.41 [0.10, 0.72] | |
| General health | iABCT | 52.29 (17.41) | 62.96 (19.33) | 60.00 (23.52) | **0.61 [0.37, 0.84]** | **0.44 [0.19, 0.69]** | |
|  | WL | 58.00 (19.61) | 62.12 (16.50) | 61.17 (22.62) | -0.21 [-0.44, 0.03] | -0.16 [-0.41, 0.09] | |
|  |  |  |  |  |  |  | |
| **Well-being**  *PHI* |  |  |  |  |  |  | |
| Total well-being | iABCT | 5.91 (1.99) | 6.43 (1.82) | 6.10 (2.07) | **0.26 [0.03, 0.48]** | -0.09 [-0.25, 0.06] | |
|  | WL | 6.32 (1.72) | 6.03 (1.87) | 5.95 (2.73) | 0.17 [0.01, 0.33] | 0.21 [0.02, 0.40] | |
| Remembered well-being | iABCT | 5.92 (2.03) | 6.48 (1.89) | 6.12 (2.16) | **0.27 [0.04, 0.50]** | -0.10 [-0.25, 0.06] | |
|  | WL | 6.36 (1.75) | 6.08 (1.94) | 5.94 (2.82) | 0.16 [0.00, 0.31] | 0.24 [0.04, 0.43] | |
| Experienced well-being | iABCT | 5.76 (2.46) | 5.78 (2.68) | 5.94 (1.92) | -0.01 [-0.29, 0.27] | -0.07 [-0.35, 0.20] | |
|  | WL | 5.87 (2.26) | 5.47 (2.30) | 6.09 (2.77) | 0.18 [-0.14, 0.49] | -0.10 [-0.41, 0.21] | |
|  |  |  |  |  |  | |  |

*Note. d, Cohen’s d; CI, Confidence Interval; iABCT, Internet Attachment-based Compassion Therapy; WL, waiting list; EQ-5D, EuroQol 5D; PHI, Pemberton Happiness Scale.*

**Table S5.** Means, standard deviations, and within-group effect sizes for secondary psychological outcomes assessed at baseline, 3-month, and 6-month follow-up in adults with chronic medical conditions participating in a two-arm parallel randomized controlled trial of Internet-Delivered Attachment-Based Compassion Therapy (iABCT).

| **Measure** | **Condition** | **M (SD)** | | | **Within-group effect size, *d* [95% CI]** | |
| --- | --- | --- | --- | --- | --- | --- |
|  |  | **Pre** | **FU (3M)** | **FU (6M)** | **Pre vs. 3M** | **Pre vs. 6M** |
| **Compassion and self-compassion**  *SOCS-S* |  |  |  |  |  |  |
| SOCS Total score | iABCT | 66.78 (11.60) | 74.52 (11.20) | 74.33 (13.07) | **0.66 [0,38, 0,94]** | **0.64 [0,42, 0,86]** |
|  | WL | 73.67 (11.81) | 73.58 (13.52) | 75.27 (14.70) | -0.01, [-0.14, 0.16] | -0.13 [-0.32, 0.05] |
| **Self-care behaviors**  *B-MSCS* |  |  |  |  |  |  |
| Physical care | iABCT | 2.79 (0.55) | 2.66 (0.78) | 2.79 (0.69) | 0.23 [-0.06, 0.52] | 0,00 [-0.25, 0.25] |
|  | WL | 2.84 (0.52) | 2.80 (0.63) | 3.20 (0.59) | -0.08 [-0.16, 0.31] | **0.68 [0.38, 0.99]** |
| Mindful relaxation | iABCT | 2.27 (0.94) | 2.44 (0.79) | 2.31 (0.73) | -0.18 [-0.49, 0.13] | 0.04 [0.30, -0.39] |
|  | WL | 2.58 (0.92) | 2.77 (1.13) | 2.73 (1.16) | -0,20 [-0.41, 0.00] | -0.16 [-0.41. 0.08] |
| Self-compassion and purpose | iABCT | 2.63 (1.06) | 3.27 (0.89) | 2.93 (0.96) | **0.60 [0.24, 0.95]** | 0.60 [0.24, 0.95] |
|  | WL | 3.06 (1.15) | 3.11 (0.94) | 3.20 (1.31) | -0.04 [-0.25, 0.17] | -0.12 [-0.30, 0.06] |
| Supportive relationships | iABCT | 3.74 (1.05) | 4.05 (0.94) | 3.92 (1.00) | **0.29 [0.02, 0.56]** | -0.17 [-0.50, 0.16] |
|  | WL | 3.59 (1.03) | 3.54 (1.16) | 3.89 (1.13) | -0.05 [-0.08, 0.18] | 0.29 [0.05, 0.53] |
| Supportive structure | iABCT | 3.16 (0.92) | 3.52 (0.66) | 3.58 (0.63) | 0.39 [0.13, 0.64] | 0.39 [0.13, 0.64] |
|  | WL | 3.35 (1.04) | 3.38 (0.89) | 3.43 (1.05) | -0.03 [-0.17, 0.11] | -0.08 [-0.26, 0.10] |
| Mindfulness | iABCT | 3.29 (1.15) | 3.69 (1.02) | 3.39 (1.04) | 0,34 [0,05, 0,64] | 0.34 [0.05, 0.64] |
|  | WL | 3.66 (1.06) | 3.67 (1.05) | 3.67 (1.09) | -0.01 [-0.20, 0.19] | -0.01 [-0.25, 0.23] |
| **Self-criticism:**  *SCRS* |  |  |  |  |  |  |
| SCRS Total score | iABCT | 26.76 (7.68) | 21.56 (8.90) | 23.11 (10.98) | **0.67 [0.40, 0.94]** | **0.47 [0.28, 0.66]** |
|  | WL | 23.40 (8.12) | 24.04 (9.53) | 23.91 (11.75) | -0.08 [-0.17, 0.02] | -0.06 [-0.17, 0.05] |
| **Symptomatology:**  *DASS-21* |  |  |  |  |  |  |
| Anxiety | iABCT | 13.32 (11.13) | 8.12 (8.72) | 9.50 (10.71) | **0.46 [0.21, 0.71]** | **0.34 [0.16, 0.52]** |
|  | WL | 12.48 (10.39) | 8.38 (8.56) | 8.73 (9.36) | **0.39 [0.16, 0.62]** | 0.36 [0,08,0.64] |
| Depression | iABCT | 18.14 (12.44) | 11.75 (13.14) | 11.83 (13.78) | **0.51 [0.26, 0.75]** | **0.50 [0.28, 0.72]** |
|  | WL | 16.05 (12.26) | 10.88 (9.42) | 10.81 (12.48) | **0. 42 [0.21, 0.62]** | **0.42 [0.19, 0.66]** |
| Stress | iABCT | 21.14 (10.22) | 13.76 (10.51) | 14.22 (11.02) | **0.71 [0.45, 0.98]** | **0.67 [0.42, 0.92]** |
|  | WL | 20.19 (10.42) | 14.62 (7.97) | 16.18 (11.88) | **0.53 [0.26, 0.79]** | 0.38 [0.11, 0.64] |
| DASS Total score | iABCT | 52.59 (31.43) | 34.42 (30.28) | 35.56 (33.68) | **0.57 [0.32, 0.82]** | **0.54 [0.34, 0.73]** |
|  | WL | 48.71 (30.41) | 34.92 (23.80) | 35.73 (29.55) | **0.45 [0.22, 0.67]** | 0.42 [0.17, 0.67] |
| **Attachment styles:**  *RQ* |  |  |  |  |  |  |
| Secure attachment | iABCT | 4.56 (2.05) | 5.08 (1.68) | 3.61 (2.48) | **0.25 [0.02, 0.48]** | 0.46 [0.09, 0.83] |
|  | WL | 4.86 (1.89) | 4.27 (1.73) | 4.09 (2.12) | 0.31 [0.05, 0.57] | 0.40 [0.19, 0.61] |
| Preoccupied attachment | iABCT | 3.70 (2.21) | 2.48 (2.12) | 2.17 (2.20) | **0.55 [0.26, 0.83]** | **0.68 [0.40, 0.97]** |
|  | WL | 3.19 (2.10) | 3.69 (1.93) | 2.36 (2.11) | 0.24 [0.03, 0.44] | 0.39 [0.06, 0.71] |
| Dismissive attachment | iABCT | 3.95 (1.80) | 3.52 (1.92) | 2.72 (2.30) | 0.24 [-0.02, 0.49] | **0.67 [0.40, 0.95]** |
|  | WL | 4.24 (1.75) | 3.69 (1.85) | 3.36 (1.75) | 0.31 [-0.02, 0.64] | 0.50 [0.12, 0.87] |
| Fearful attachment | iABCT | 3.81 (2.20) | 2.40 (2.00) | 2.83 (2.43) | **0.63 [0.36, 0.91]** | **0.44 [0.20, 0.68]** |
|  | WL | 3.17 (2.13) | 2.65 (2.02) | 2.64 (2.62) | 0.24 [-0.01, 0.49] | 0.25 [0.00, 0.49] |
| **Social support:**  *MOS-SSS* |  |  |  |  |  |  |
| MOS-SSS Total score | iABCT | 74.60 (17.05) | 77.33 (16.16) | 79.00 (18.33) | -0,16 [-0.38, 0.06] | 0.25 [0.05, 0.46] |

*Note.* d, Cohen’s d; CI, Confidence Interval; Pre, Pre-assessment; Post (3M), 3-month follow-up; FU (6M), 6-month follow-up; iABCT, Internet Attachment-based Compassion Therapy; WL, waiting list; Mean dif., mean differences; SOCS-S = Sussex-Oxford Compassion for the Self Scale, B-MSC = Mindful Self-Care Scale - Brief version; SCRS = Self-critical rumination Scale, DASS-21 = Depression, Anxiety and Stress Scale; RQ = Relationships Questionnaire, MOS-SSS = Medical Outcomes Study-Social Support Survey.

# Participant’s opinion. Reasons for dropping out or not participating in CBI intervention and characteristics to improve engagement

This section provides comprehensive qualitative analysis to complement the main findings reported in Section 3.7. Complete frequency analyses, additional barriers not reported in the main text, and detailed participant responses are presented below.

A total of 74 participants who dropped out from the study or decided not to participate were contacted to complete the semi-structured interview. Responses were completed by a total of 34 participants (21 of the non-participation group and 13 participants of the drop-out group). This represents a 46% response rate (34/74). All individuals who either did not complete baseline assessment (n=53) or dropped out after baseline (n=21) were contacted via email with an invitation to complete the interview; participation was voluntary and no incentives were provided. One researcher (MM) conducted initial open coding to identify facilitators and barriers. Codes were then reviewed by a second researcher (MDV) with experience in qualitative analysis. Discrepancies were discussed until consensus was reached.

## Detailed barriers to Participation

Regarding barriers, frequency analyses revealed that the most frequent reasons to not participate in the study were not feeling motivated (n= 19, 90.5%), the presence of too many steps to access to the intervention (n= 14, 66.7%) or not being interested in the program (n= 13, 61.9%) or the scientific research (n= 12, 57.1%). Other reasons were related to missing the presence of a therapist (n= 9, 42.9%), not having enough time (n=9, 42.9%) or considering that the assessment part was too long or with too many questionnaires (n= 9, 42.9%). Other reasons mentioned by participants could be seen in Table S6.

**Table S6.** Reasons for not participating.

| **Reasons to not participate** | **N= 21** | **% sample** |
| --- | --- | --- |
| Improvement of emotional state | 2 | 9.5 |
| Missed the presence of a therapist | 9 | 42.9 |
| I did not feel motivated | 19 | 90.5 |
| I was not interested in the program | 13 | 61.9 |
| I was not interested in the science research | 12 | 57.1 |
| The platform was not what I expected | 6 | 28.6 |
| Problems with access | 7 | 33.3 |
| Preference for another electronic device (smartphone or tablet) | 0 | 0 |
| There were too many steps for accessing | 14 | 66.7 |
| The assessment was too long, with too many questionnaires | 9 | 42.9 |
| Some situation related to COVID-19 | 0 | 0 |
| Other:  Time | 9 | 42.9 |

## Detailed drop-out Analysis

On the other hand, results show that the most frequent reasons for dropping out from the intervention were related with needing the physical contact with a therapist (n= 12, 92.3%), need for more monitoring or support (n= 8, 61.5%), feeling that there were elements from the intervention that were too demanding, tiring or repetitive (n= 8, 61.5%), or feeling that the platform was not what they expected (n=7, 53.8%). Other less frequently mentioned reasons were that the platform was not likeable for them (n= 6, 46.2%), or that the program required too much time (n= 6, 46.2%) (see Table S7 for more details).

**Table S7.** Reasons for dropping out of the treatment.

| **Reasons for dropping out** | ***N*= 13** | **%** |
| --- | --- | --- |
| Improvement of emotional state | 3 | 23.1 |
| The program was not helping | 1 | 7.7 |
| The program was not what I expected | 2 | 15.4 |
| Need for physical contact with a therapist | 12 | 92.3 |
| Need for more monitoring | 8 | 61.5 |
| Need for greater restraint | 0 | 0 |
| I did not feel motivated | 3 | 23.1 |
| I was not interested in the program | 0 | 0 |
| The platform was not what I expected | 7 | 53.8 |
| The platform format was not likeable to me | 6 | 46.2 |
| Problems with access | 1 | 7.7 |
| Preference for another electronic device (smartphone or tablet) | 2 | 15.4 |
| The program was difficult to adapt to daily life. | 2 | 15.4 |
| Too much time | 6 | 46.2 |
| There were elements too demanding, tiring or repetitive (e.g. questionnaires, exercises or practices) | 8 | 61.5 |
| The program was too long | 2 | 15.4 |
| Some situation related with COVID-19 | 0 | 0 |
| Other:  Not being constant and tendency to not finish what I started | 1 | 7.7 |

## Detailed Facilitators and Desired Improvements

Regarding facilitators, improvements such as incrementing the presence of a therapist (79.4%), an easier and more attractive design (73.5%), a shorter program (55.9%) or having more interaction (55.9%), were the most frequently mentioned for both groups. Other important improvements were related to a more tailored and specific program (32.4% and 23.5% respectively) or including functionalities to interact with others (32.4%) (see Table S8 for more details).

**Table S8.** Facilitators of CBI.

| **Facilitators** | ***N*= 34** | **% sample** |
| --- | --- | --- |
| A shorter program (brief) | 15 | 55.9 |
| Different design (easier and attractive) | 25 | 73.5 |
| Different format (e.g. app for smartphone) | 3 | 8.8 |
| Tailored program | 11 | 32.4 |
| A more specific and targeted program for my problem | 8 | 23.5 |
| More presence of a therapist | 27 | 79.4 |
| Completely face-to-face intervention | 3 | 8.8 |
| More interaction | 15 | 55.9 |
| Functionalities to interact (e.g. chats, forums) | 11 | 32.4 |
| Other:  technical problems | 2 | 5.9 |

# Qualitative analyses of participants’ opinions on improvements and missed aspects of the program.

Results from the qualitative thematic analysis were organized into four domains including: support, program improvements, aspects of initial assessment and access, and platform and web facilitators. A total of 15 categories are included in the domains (see Table S9 for details).

## Support

This domain refers to the participant’s wish to establish contact with some therapist or interacting with other peers that could help them and support them along the intervention. A total of 4 categories related to the support emerged from the analysis: therapist support, support not specified, support from other peers and support from guides. Improvements included in this domain were the most mentioned by participants, representing 29.75% of the total. Specifically, support from therapist was the most mentioned improvement within the domain (47.2%), followed by no specified support (30,5%), support from other peers (16.67%), and support from a guide (5.56%).

## Program

This domain contains participant’s perceived improvements regarding the different characteristics of the iABCT program such as the content or the amount of information among others. Comments from participants regarding characteristics of the program were classified into 4 categories: personalization, length, difficulty level and dynamism. Most comments were made in relation to the length of the program (40.63%), referring to the wish of participants of a shorter program. Other aspects mentioned by participants referred to a more personalized and adapted program (21.88%), and an easier (18.75%) and more dynamic program (18.75%).

## Aspects of initial assessments and access

Aspects mentioned in this domain were related to participant’s opinion of the different assessments and the procedure to access the web platform and start with the intervention. Three categories were included in this domain containing comments regarding participant’s wish for shorter assessments (33.33%), a reduced number of assessments (22.22%) and less steps to access the platform and start with the intervention (44.44%).

## Web platform

This domain was characterized by participants’ comments on different web platform features (i.e. website design) or different aspects regarding technology. A total of 4 categories related to the web platform emerged from the analysis: intuitive web (28.57%), technical aspects (20%), accessibility (11.43%) and system design (40%). Most of the participants’ desires mentioned a more attractive and user-friendly web platform.

**Table S9.** Domains, categories and examples of qualitative thematic analysis.

| **Domain** | **Category** | **Example** | **Nº of units** | **% of the domain** | **% total** |
| --- | --- | --- | --- | --- | --- |
| **Support and interaction** | No specified human support | *“More support and interaction”*  *“Someone that could help me and support me”* | 11 | 30.5% | 29.75% |
|  | Therapist | *“Increased therapist presence or include sessions in which a therapist is present”*  *“I missed the help or support of a professional”* | 17 | 47.2% |  |
|  | Other peers | *“To be able to talk to other people”*  *“I wish I could have talked to other patients”* | 6 | 16.67% |  |
|  | Guide | *“Some lessons via zoom or similar to increase commitment and involvement”*  *“Guidance to understand some aspects of the program”* | 2 | 5.56% |  |
| **Program** | Personalization | *“A more personalized program adapted to my disease”*  *“More personalization. I felt that the program was not adapted to my condition”.* | 7 | 21.88% | 26.45% |
|  | Length | *“A shorter program, with less modules”*  *“The program seemed very long, I couldn't find the time to accommodate it”* | 13 | 40.63% |  |
|  | Difficulty level | *“An easier program”*  *“The program was quite complicated and tedious at certain points”* | 6 | 18.75% |  |
|  | Dynamism | *“A more dynamic and motivating program”*  *“The program was repetitive and boring”* | 6 | 18.75% |  |
| **Aspects of initial assessments and access** | Shorter assessments | *“The assessments were very long”*  *“Fewer or at least shorter questionnaires”* | 6 | 33.33% | 14.88% |
|  | Number of assessments | *“Reduced number of questionnaires”*  *“Fewer assessments”* | 4 | 22.22% |  |
|  | Less steps to start | *“Reduce tasks to be performed before the intervention”*  *“There were too many steps to access”* | 8 | 44.44% |  |
| **Web platform** | Intuitive web | *“A more user-friendly website”*  *“A more intuitive web platform”* | 10 | 28.57% | 28.93% |
|  | Technical aspects | *“The program did not allow access, there were a lot of errors”.*  *“The website did not run properly”* | 7 | 20% |  |
|  | Accessibility | *“Easier access”*  *“Better access to the platform”* | 4 | 11.43% |  |
|  | System design | *“I found the format of the website a bit old-fashioned and not very attractive”*  *“A more visually appealing platform”* | 14 | 40% |  |
